# Dynamic regulation of BACH2 enhances CAR T cell function

**DOI:** 10.1016/j.omton.2026.201164

**Published:** 2026-03-07

**Authors:** Tien-Ching Chang, John M. Warrington, Nathan Singh

**Affiliations:** 1 Center for Genetic and Cellular Immunotherapy, Washington University School of Medicine, St Louis, MO, USA

## Main text

Chimeric antigen receptor (CAR) T cells have fundamentally altered the treatment landscape for hematologic malignancies.^1^^,^^2^ To create this therapy, a patient’s own T cells are engineered *ex vivo* to constitutively express a CAR and then infused back into the patient. CARs consist of an extracellular target (antigen)-recognition domain linked to an intracellular T cell signaling domain that combines 4-1BB or CD28 with CD3ζ. Upon extracellular antigen binding, CARs transmit T cell activation signals across the membrane, initiating target cell killing, cytokine secretion, and T cell proliferation. Despite their remarkable ability to generate stable remissions with just a single dose, CAR T cells still fail most patients.

Antigen-independent “tonic” signaling has been pointed to as a cause of this failure.^3^^,^^4^^,^^5^^,^^6^^,^^7^^,^^8^ Previous studies have shown that tonic signaling can impair function by driving premature T cell exhaustion in CD28-containing CARs; however, our previous data demonstrate that tonic signaling of 4-1BB-containing CARs paradoxically enhances T cell fitness in CARs.^9^ This functional divergence offers a window into the molecular circuitry regulating engineered T cell fitness and failure. We sought to elucidate the molecular mechanisms by which 4-1BB tonic signaling confers superior functionality. Our work demonstrates that the transcriptional regulator BACH2 is a dose- and time-dependent regulator of T cell function that can be controlled to enhance the long-term efficacy of exhaustion-prone-CARs in multiple tumor models.^10^

We first undertook transcriptomic and epigenomic analyses of tonically signaling, 4-1BB-containing, CD22-targeted CAR T cells, which identified both increased expression of BACH2 target genes and increased accessibility at genomic BACH2 binding sites. This was accompanied by increased BACH2 transcript and protein counts, all occurring during product manufacturing in the absence of antigen engagement. BACH2 is well known as a regulator of lymphocyte fate, promoting quiescent, stem-like, and memory programs in both B and T cells,^11^^,^^12^ suggesting a mechanism by which tonic 4-1BB may prevent terminal differentiation by instead supporting memory-like states.

To overcome tonic CD28 signaling-driven CAR T cell exhaustion, we transgenically overexpressed BACH2 in tonically signaling CD22-targeted CARs containing CD28 domains. BACH2 overexpression reduced expression of exhaustion-associated proteins, enhanced cytotoxicity, and shifted T cells toward central memory lineages, similar to the phenotype of T cells expressing tonically signaling 4-1BB-based CARs. CRISPR-mediated disruption of BACH2 in tonic 4-1BB CAR T cells resulted in restrained memory differentiation and reduced antitumor function. These gain- and loss-of-function experiments established BACH2 as a central transcriptional regulator that prevents exhaustion, promotes memory, and enhances short-term function. Using chronic antigen stimulation models^13^^,^^14^ to investigate long-term CAR T cell function, we found that constitutive BACH2 overexpression ultimately limited long-term CAR T cell expansion and cytotoxicity. Although BACH2-overexpressing cells maintained memory phenotypes, these cells failed to efficiently transition into effector states required for prolonged function. Mechanistically, CUT&RUN analysis demonstrated that high BACH2 expression reduced binding of the factor cJUN, reflecting an antagonism of AP-1-driven effector programs. To resolve the conflict between preventing exhaustion and promoting long-term function, we adapted a tunable protein expression system that allowed quantitative control of BACH2 using a degradation domain. By adding the antibiotic trimethoprim (TMP), this domain permitted high BACH2 expression (the degron “ON” state); withdrawing TMP promoted BACH2 degradation (the degron “OFF” state). This system, however, did not result in complete degradation of BACH2 and remained partially leaky, meaning the “OFF” state was actually “BACH2 low.” Using this tunable approach, we found that BACH2 “ON” (high BACH2) mimicked the memory-locked state of classical BACH2 overexpression, committing cells to quiescent states even when expressed at high levels only during manufacturing. In contrast, BACH2 “OFF” (or rather, low) cells did not exhaust, transited to effector states, and significantly enhanced the long-term function of exhaustion-prone CAR T cells. We confirmed this in *in vitro* and *in vivo* models of human leukemia. We next tested this approach using a highly toxic, tonically signaling CAR targeting the neuroblastoma antigen GD2.^15^^,^^16^ This CAR has been the paradigm to study tonic CD28-driven exhaustion. We found that BACH2 expression again prevented exhaustion and enhanced function, but in contrast to the CD22 CAR model, this CAR required higher levels of BACH2 to antagonize exhaustion signaling. Lastly, we interrogated clinical CD19 CAR T cell products to assess any similar relationship between BACH2 activity and therapeutic efficacy. We found that products with higher BACH2 signatures were much more effective and that this signature was itself a strong predictor of survival.

Ultimately, these studies establish that BACH2 serves as a central rheostat controlling rest, activation, and exhaustion programs in engineered T cells and that dynamic regulation of BACH2 can serve as a tunable platform to overcome central barriers facing T cell therapies.

## Declaration of interests

T.-C.C. and N.S. have submitted patent applications related to this work. J.M.W. is named on patents related to CAR T cell therapy that are managed by the Children’s Hospital of Philadelphia. N.S. has patents related to CAR T cell therapy, some of which are licensed to Novartis, and all of which are managed by either the University of Pennsylvania or Washington University School of Medicine. N.S. is a member of the Board of Directors for Phoreus Bio and is a co-founder of Defiance Therapeutics.
